# Extracellular Vesicles in Bacteria, Archaea, and Eukaryotes: Mechanisms of Inter-Kingdom Communication and Clinical Implications

**DOI:** 10.3390/microorganisms13030636

**Published:** 2025-03-11

**Authors:** Maria Di Naro, Giulio Petronio Petronio, Farwa Mukhtar, Marco Alfio Cutuli, Irene Magnifico, Marilina Falcone, Natasha Brancazio, Antonio Guarnieri, Roberto Di Marco, Daria Nicolosi

**Affiliations:** 1 Department of Medicina e Scienze della Salute “V. Tiberio”, Università degli Studi del Molise, 86100 Campobasso, Molise, Italy; 2 Aileens Pharma S.r.l., 20834 Nova Milanese, Monza and Brianza, Italy; 3 Department of Drug and Health Sciences, Università degli Studi di Catania, 95125 Catania, Sicily, Italy

**Keywords:** extracellular vesicles, bacteria-derived extracellular vesicles, archaea EVs, cell communication, interdomain communication, clinical applications of EVs

## Abstract

Living organisms must adapt and communicate effectively in their environment to survive. Cells communicate through various mechanisms, including releasing growth factors, chemokines, small bioactive molecules, and cell–cell contact. In recent years, a new and sophisticated cell communication mechanism based on extracellular vesicles (EVs) has been described in all three domains of life: archaea, bacteria, and eukaryotes. EVs are small, bilayer proteolipid vesicles released by cells into the extracellular space. This review aims to analyze and compare the current literature on bacterial, archaeal, and eukaryotic EVs and their possible clinical applications. This framework will address three key points: (a) The role of EVs in bacteria, eukaryotes, and archaea. (b) What is the impact of EVs in archaea on disease? (c) How archaea use EVs to communicate with other domains (bacteria/eukaryotes).

## 1. Introduction

Over the past twenty years, the scientific interest in extracellular vesicle (EV) projects has increased, and the number of studies including the term “extracellular vesicles” has exploded in the scientific literature. In 2000, PubMed referred to 2492 studies, which, over twenty years, surged to above 43,500 results.

Extracellular vesicles, characterized as spherical, bilayered proteolipids with an average diameter of 20–500 nm, are produced across all domains of life as a conserved phenomenon in eukarya, archaea, and bacteria [1,2,3]. It is widely known that no cell lives isolated in our body, but instead a complex communication network between cells guarantees the homeostasis of tissues and organs. All cells communicate and interact with each other through a phenomenon known as cell signaling.

In some cases, this cell-to-cell communication is direct; in other cases, the cells can communicate at a distance: signaling cells produce signal molecules, which are recognized by a target cell through receptor proteins, producing other intracellular signals. This process, which encodes the information the extracellular messenger carries into intracellular changes, is known as signal transduction. The signals produced can have short- or long-term effectiveness. Another cell–cell communication mechanism is based on releasing these heterogeneous groups of lipoprotein vesicles, i.e., the extracellular vesicles (EVs), which act as vehicles of intercellular information [4].

EVs serve as key mediators of intercellular communication by transporting a diverse array of molecular cargoes, which they deliver to recipient cells through membrane fusion and/or endocytosis in eukaryotes, thereby influencing cellular physiology [1]. EVs have been implicated in various biological processes, including the stress response, intercellular competition, lateral gene transfer (via RNA or DNA), pathogenicity, and detoxification. Their role in human pathologies and aging has recently attracted significant interest.

Several reviews have explored specific aspects of EV function, such as horizontal gene transfer (HGT) [5], immune response modulation [6], aging [7], RNA cargo composition [8], cancer progression [9], and EV–virus interactions [10,11]. While extensive studies have focused on bacterial EVs, particularly outer membrane vesicles (OMVs) [9,11,12,13] others have investigated eukaryotic EVs, with a particular emphasis on exosomes [14,15,16,17,18]. However, despite the growing interest in EVs, research on archaeal EVs remains limited [1,15]. In this review, we will explore the EVs produced by bacteria and eukaryotes, focusing mainly on the EVs produced by archaea [19,20] and on their possible role as a source of communication between the different kingdoms.

## 2. Extracellular Vesicles: More than Just Cellular Trash

EVs were identified initially as a cellular byproduct of cleanup and clearance. However, they are essential to various biological processes because they are critical in cell-to-cell communication. Recent developments in the EV field have contributed to a remarkable proliferation of evidence, discovering that these carrier vesicles are not mere cellular waste but essential for cellular functions [21]. In particular, EVs released by cells to the extracellular space transfer the cellular functional load between cells, ultimately altering their recipient cells’ functionality and facilitating various biological processes as mediators of intercellular communication [22].

The International Society for Extracellular Vesicles (ISEV) uses a definition that refers to EVs as ’particles released by cells that consist of a membrane-bound bilayer, exclude nucleic acids needed for replication, and are not capable of replication’ [23,24]. Thus, despite numerous studies investigating EVs, the formation mechanism of EV production is still unclear. Although we do not know whether EV biogenesis is homologous in the three domains of life or whether different formation mechanisms exist, our current understanding of EV biogenesis mechanisms might not be connected or evolved in any way. Moreover, several studies have been conducted in the last years to better explain the biogenesis of EV production in bacteria [25,26], eukaryotes [27], and archaea [28]. Therefore, more information is required to understand how complex mechanisms of EV production work across the three domains of life.

## 3. Archaeal EV

In archaea, EVs exhibit intriguing similarities to a Gram-negative bacteria’s outer membrane vesicles (OMVs) [29]. Like in bacteria, the archaea cytoplasm is enclosed by a cytoplasmic membrane predominantly comprising glycerol phosphate phospholipids, albeit with subtle variations in lipid constituents. Furthermore, most archaea cell membranes are proteinaceous layers, or S-layers, that contribute to cell shape and protection, facilitating their colonization of extreme environments [30].

The composition and structure of the S-layer vary among different archaeal species: it is often composed of a single protein or glycoprotein, forming a pseudo-crystalline structure that can be associated with the cytoplasmic membrane; in all the species of Methanothermus and Methanopyrus, a pseudo murein polymer, akin to bacterial peptidoglycan, forms a secondary cell wall beneath the S-layer; Methanosarcina species present an S-layer along with an optional layer of methanochondroitin; and notably, H. walsbyi strain HBSQ001 is covered by two S-layers [31].

Some members of *Thermoplasma, Halococcus, Methanobrevibacter, Natronococcus, Methanosphaera, Ignicoccus* genera, and *Thermosphaera* aggregates lack an S-layer structure. Nevertheless, they thrive in harsh environments with high temperatures and low pH. The preservation of cellular integrity probably relies on glycocalyx, membrane-associated glycoproteins, or lipoglycans. In some species of the Ignicoccus genus, a unique feature is observed: a double membrane with distinct inner and outer cellular membranes, resembling the compartmentalization seen in Gram-negative bacteria [32,33,34,35].

It is essential to highlight that all archaea displaying a double membrane-based cell wall interact closely with other archaea, bacteria, or their eukaryotic host [36].

Although some archaea possess rigid cell walls, like those of the orders *Methanobacteriales, Methanopyrales* (with their pseudomurein layer), and the genus *Halococcus* (with its complex heteroglycan wall), the majority have a more flexible cell wall structure. The lack of a rigid cell wall might facilitate the production and release of extracellular vesicles and nanotubes [35,37,38].

Another relevant aspect related to the biogenesis of the EV is the cell division system. Most Euryarchaeota encode for a bacterial-like FtsZ-based system, suggesting a division mechanism similar to bacteria [39,40].

Conversely, *Crenarchaeotes* inhabiting high-temperature environments lack FtsZ but divide through binary fission. Interestingly, these organisms utilize an alternative cell division mechanism analogous to the eukaryotic: the Endosomal Sorting Complexes Required for Transport-III (ESCRT-III) and VPS4 ATPase. The ESCRT apparatus plays crucial roles in various intracellular membrane processes in eukaryotes, including vesicle formation [20].

Other archaea species such as *Thaumarchaeota* and the *Euryarchaeota*, members of the orders *Thermococcales* and *Thermoplasmatales*, encode components of both systems. It is still unknown whether they cooperate or perform distinct functions. *Thermoproteales* lack both FtsZ and ESCRT-III. It has been proposed that they divide through the action of the crenactin cytoskeleton and the actin-related arcadins [39,41,42,43].

Most studies on archaea EV production have been performed on the phyla *Crenarchaeota* (order Sulfolobales) or *Euryarchaeota* (order *Thermococcales* and class *Halobacteria*) [44].

Based on their biogenesis and taxonomic origin, archaeal EVs have been classified as crenarchaeotal AEVs (C-AEVs) produced via the archaeal ESCRT machinery, and euryarchaeotal AEVs (E-AEVs) produced via cell membrane blebbing [45].

Regarding EV production from the *Crenarchaea* phylum, most studies have been conducted on organisms belonging to the Sulfolobales, which have shown EVs with diameters ranging from 90 to 230 nm, coated with an S-layer, and associated with antimicrobial proteins known as ’sulfolobicins’. These proteins effectively inhibit the growth of related Sulfolobus species and represent a novel class of antimicrobial proteins with no homology to others. Studies on *S. islandicus* and *S. tokodaii* have revealed that a significant portion of sulfolobicin activity in the medium fraction is linked to membrane vesicles. Although the association with EVs is not necessary for antimicrobial activity, the fact that most of the activity resides in the membrane-associated fraction suggests that EVs serve as an efficient vehicle for delivery [46,47]. Proteomic analysis of the vesicles released by Sulfolobus sp. revealed that they contain the S-layer proteins, and similarly to eukaryotic exosomes, the homologous-to-eukaryotic ESCRT-III proteins together with an archaeal VPS4-like ATPase.

Among the Euryarcheota, most studies focused on EV production rely on the order *Thermococcales*. Conversely, from *Crenarcheota*, the genome of *Euryarchaeota* does not encode ESCRT-III homologs [41]. Hence, the mechanism of EV production in this phylum is likely to be different from what is observed in *Sulfolobales*. Most strains of *Thermococcales* produce EVs of about 50–150 nm. The biochemical characterization of purified EVs from two species of Thermococcales, *T. kodakarensis* and *T. gammatolerans*, revealed that the protein and lipid profiles of EVs and parent cell membranes have a similar composition [48]. The mechanism behind the EVs in these archaea is still unknown. One hypothesis is that the production by budding from the cell membrane can be driven by some proteins involved in cell division, similar to ESCRT proteins in Crenarchaeota. Another hypothesis is that EV formation depends on proteins involved in polar lipids’ biosynthesis, especially those that modify the polar head groups [20].

The vesicles might be produced as a response to stress. This idea is supported by the presence of the FHA protein and vWA-containing proteins, which likely work together in a signaling pathway. Similar protein domains in other organisms, like eukaryotes, form protein clumps (aggregates) and interact with proteins. Additionally, they play a role in various stress responses. These findings suggest a similar function for these proteins in archaea [49,50,51].

Interestingly, vWA domain proteins also play a role in extracellular matrix formation and cell adhesion, raising the possibility that the vesicles in archaea could also be involved in cell adhesion and/or biofilm formation [52,53].

### 3.1. EVs as a Communication Tool That Transfer Material from Cell to Cell

In archaea, particularly within the order *Thermococcales* (phylum *Euryarchaeota*), the DNA associated with these vesicles exhibits enhanced stability even under the extreme temperatures characteristic of hyperthermophiles. Additionally, it shows increased resistance to micrococcal nuclease compared to free DNA. This suggests that the association of extracellular DNA with vesicles could protect it from degradation.

The EVs generated by the budding process can fuse with recipient cells to safely deliver their content. Hence, the EVs generated by hyperthermophile Thermococcales represent an important mechanism for cell adaptation to the environment, acting as an important means of communication to safely transfer proteins, peptides, nucleic acids, other macromolecules, and metabolites from cell to cell. In this scenario, beyond the role of protection and carrier of nucleic acids, in *Euryarchaeota*, ord. *Halobacteriales* and *Thermococcales*, EVs can facilitate the recombination between viral, plasmid, and/or cellular chromosomes [48,54,55,56] (Table 1).

Similarly to Euryarchaeota, the EVs produced by Crenarchaea of the order Sulfolobales, which represent the majority of inhabitants of terrestrial acidic hot springs, can transport nucleic acids, suggesting that horizontal gene transfer (HGT) might be a general property across the archaea. For such a peculiar mechanism in which the EVs mediate the HGT, the term “vesiduction” has been coined to differentiate it from the three traditional main mechanisms of HGT: natural transformation, conjugation, and transduction. The vesiduction process entails the following stages: (i) the release of an EV harboring DNA from a donor cell, (ii) the migration of the EV through the surrounding environment, (iii) the adhesion of the EV to the surface of a recipient cell, (iv) the DNA conveyance into the cytoplasm, and (v) the gene acquisition. The analysis of EVs produced by *Haloarchaea volcanii* and other *Haloarchaea* shows that EVs are associated with RNA in a conserved process. It has been proposed that producing EVs enclosing RNA is an active process through which *Archaea* exploit the EVs as a communication tool to influence the gene expression population-wide, as proposed for other bacteria [56,57,58,59].

Several studies have shown that EVs can play a role in detoxification in eukaryotes and bacteria. Similarly, this mechanism has been observed in archaea. Some *Thermococcus* species, such as *T. prieurii* and *T. kodakaraensis*, produce dark vesicles that accumulate excess sulfur and are released outside the cell, suggesting that EVs can act as a tool to eliminate excess toxic substances [60].

Another possible role for EVs is related to using EVs as a source of nitrogen and carbon to support heterotrophic cell growth. This is likely to be crucial in natural settings where nutrients are scarce. Such a process is observed in *S. islandicus*, and is similarly observed in *Prochlorococcus*, a marine cyanobacterium, which is known to release EV-containing lipids, proteins, and nucleic acids so that *Prochlorococcus’* vesicles can support the growth of heterotrophic bacterial cultures [20,61].

DPANN is an archaeal superphylum that includes members with extremely small cell and genome sizes and limited metabolic capabilities. The limited catabolic and anabolic capabilities make many DPANN members dependent on symbiotic interactions with other organisms [62,63,64,65].

The symbiotic interaction is well described in the association of *Nanoarchaeum equitans* (a member of the *Nanoarchaeota*) with its host, the diderm crenarchaeon *Ignicoccus hospitalis*. *N. equitans’* genome is the smallest of all archaea known today; it lacks genes for the biosynthesis of lipids, amino acids, nucleotides, and cofactors [66]. Experimental evidence shows that lipids and amino acids are transported from *I. hospitalis* to *N. equitans*.

The membrane vesicles produced by *I. hospitalis’* cells might be involved in the transport of metabolites through the periplasm to the outer membrane, where they are finally released to the symbiont *N. equitans*.

Additionally, vesicles play a role in the *I. hospitalis*, dividing cells by delivering constituents to a new outer membrane [67,68,69].

### 3.2. Structural Differences Between Archaeal EVs and Other Domains

**Figure 1:**
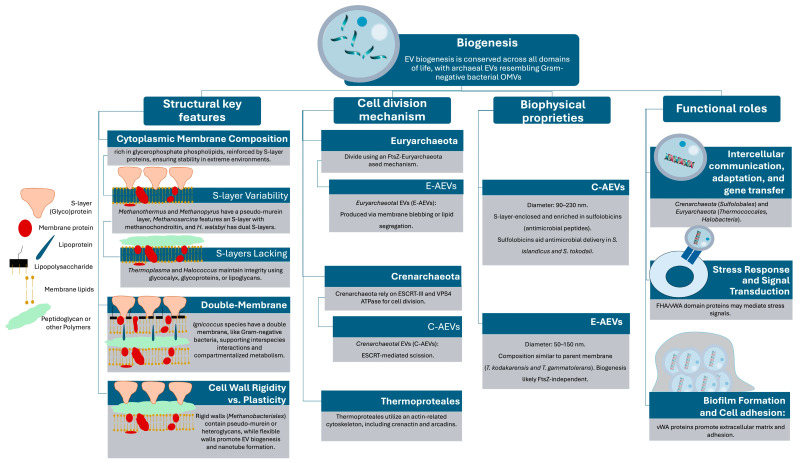
Extracellular vesicle (EV) biogenesis in archaea. Structural features, cell division mechanism, biophysical properties, and functional roles [20,30,31,32,33,34,35,36,37,38,39,40,41,42,43,44,45,46,47,48,49,50,51,52,53,54].

Archaeal extracellular vesicles (EVs) exhibit unique structural features that set them apart from their bacterial and eukaryotic counterparts. Their distinct lipid composition, surface architecture, and biogenesis mechanisms reflect the extreme environments archaea inhabit and their evolutionary divergence from bacteria and eukaryotes. Key differences include the presence of ether-linked lipids, the frequent association with S-layer proteins, and vesicle formation mechanisms that, in some cases, parallel those of eukaryotic exosomes rather than bacterial vesicles [29,30]. These features contribute to the remarkable stability and functional versatility of archaeal EVs, enabling them to participate in interdomain communication, stress adaptation, and genetic exchange (Figure 1).

Archaeal EVs share some similarities with bacterial outer membrane vesicles (OMVs), particularly in terms of their size range and their role in horizontal gene transfer (HGT) [44]. However, their lipid composition and biogenesis mechanisms are notably distinct. Unlike bacterial EVs, which are derived from phospholipid bilayers composed of ester-linked fatty acids, archaeal EVs contain ether-linked lipids that provide enhanced stability in extreme environments such as high temperatures, high salinity, and low pH [30,36,37,38]. Additionally, many archaeal EVs are associated with an S-layer, a paracrystalline proteinaceous structure that influences vesicle stability and may contribute to selective cargo loading [31].

One major distinction between archaeal and bacterial EVs is their biogenesis mechanisms. In Gram-negative bacteria, OMVs bud from the outer membrane, while Gram-positive bacteria, despite lacking an outer membrane, produce cytoplasmic membrane vesicles (CMVs) that must traverse the thick peptidoglycan layer [32,33,34,35,36,37,38]. In contrast, archaeal EVs originate either through budding of the cytoplasmic membrane (as seen in Euryarchaeota) or via ESCRT-III–dependent scission (as in Crenarchaeota) [20,39,40,41,42,43,44,45,46,47,48]. The latter mechanism is reminiscent of eukaryotic exosome formation, suggesting an evolutionary connection between archaea and eukaryotic vesicular trafficking systems [4].

When comparing archaeal EVs to eukaryotic vesicles, structural complexity is another defining factor. Eukaryotic EVs, particularly exosomes, are characterized by their endosomal origin and the presence of sophisticated lipid and protein sorting mechanisms [4]. Unlike archaeal EVs, which primarily serve in stress adaptation and interdomain communication, eukaryotic vesicles play additional roles in immune modulation, tissue remodeling, and even tumor progression [23].

Beyond these structural differences, the functional roles of archaeal EVs appear to be more closely aligned with bacterial vesicles than with eukaryotic exosomes. For instance, archaeal EVs frequently contain genetic material, including plasmids and viral DNA, which suggests a significant role in horizontal gene transfer (HGT) [44]. Additionally, studies on Haloarchaea suggest that their vesicles may participate in biofilm formation, a function commonly observed in bacterial OMVs [49,50,51,52,53].

## 4. Bacterial Extracellular Vesicles (BEVs)

Both Gram-negative and Gram-positive bacteria produce extracellular vesicles (EVs), collectively termed bacteria-derived EVs (BEVs), through distinct biogenesis mechanisms, with unique compositions and functions [29,45]. BEVs play crucial roles in pathogenesis [70], interspecies and interkingdom communication [71,72], stress tolerance [73], horizontal gene transfer [74], immune stimulation [75], and potential applications in cancer and infectious disease treatment and diagnosis [29] (Table 2).

BEVs encompass various subtypes, including membrane vesicles (MVs), which differ in origin and release mode. In Gram-negative bacteria, these include outer membrane vesicles (OMVs), outer-inner membrane vesicles (O-IMVs), explosive outer membrane vesicles (E-OMVs), and in Gram-positive bacteria, cytoplasmic membrane vesicles (CMVs) and tube-shaped membranous structures (TSMSs) [26,45]. OMVs, first identified in the 1960s [76], range from 10 to 300 nm and originate from the outer membrane, containing periplasmic components. From these first studies, several other studies were performed in the following years, confirming that they are produced across diverse environments, including planktonic cultures, biofilms, eukaryotic cells, and mammalian hosts. Non-pathogenic bacterial OMVs share functions with other EVs, facilitating cellular communication, surface modifications, and component elimination [13,28].

Despite extensive studies, OMV biogenesis remains incompletely understood, with three primary models proposed: (i) loss or relocation of covalent bonds between the outer membrane (OM) and peptidoglycan (PE), allowing OM expansion and vesiculation; (ii) accumulation of peptidoglycan or protein fragments in the periplasmic space, increasing turgor pressure and membrane detachment; and (iii) enrichment of curvature-inducing molecules, such as B-band polysaccharide LPS and quinolone PQS in *Pseudomonas aeruginosa* [77]. For example, in *P. aeruginosa*, which possesses two distinct LPS species, LPSs in the A (neutral) and B (charged) bands, the insertion of Pseudomonas quinolone signal (PQS) into the outer leaflet of the OM can increase membrane curvature and lead to increased OMV production with elevated PQS levels. However, since PQS is produced only by *P. aeruginosa*, this model is limited to this species and, therefore, not applicable to others. While PQS enhances OMV formation in *P. aeruginosa*, this model is species-specific and not universally applicable [78].

Gram-negative bacteria also produce O-IMVs, which transport cytoplasmic materials like DNA and ATP [29], likely through mechanisms similar to OMVs [79]. Additionally, E-OMVs result from phage-mediated lysis [80,81]. CMVs are produced by Gram-positive bacteria and are derived from the cytoplasmic membrane. His vesicles have a single cell membrane surrounded by a thick, rigid cell wall, have a 20–400 nm diameter, and contain substances from the cytosol. In contrast, Gram-positive bacteria generate CMVs, derived from the cytoplasmic membrane and enclosed within a thick cell wall, typically ranging from 20 to 400 nm in diameter and containing cytosolic substances [29]. TSMSs, the latest type of BEV, known as nanotubes, nanowires, or nanopods, have an average tube width of 50–70 nm and are produced by Gram-positive and Gram-negative bacteria. TSMSs function as intercellular bridges facilitating molecular exchange between cells [26,80,82].

Gram-positive bacteria, characterized by a thick peptidoglycan wall, have been ignored for decades, and an understanding of their biogenesis and interaction with the host cell is still under development. For decades, Gram-positive EV biogenesis was overlooked due to the thick peptidoglycan wall barrier. Two hypotheses explain how CMVs traverse this barrier: (i) turgor pressure forces CMVs through wall pores after membrane budding, and (ii) EV-associated or EV-released enzymes, such as penicillin-binding proteins (PBPs) and autolysins (cell wall proteins), degrade peptidoglycan locally [83]. Studies support the enzymatic hypothesis, with *S. aureus* EVs exhibiting peptidoglycan-degrading enzymes [83], *B. subtilis* expressing a prophage-encoded endolysin [82], and *S. aureus* producing autolysins and phenol-soluble modulins that facilitate CMV formation [84]. The genetic pathways that lead to the release of Gram-positive CMVs have still not been studied. The genetic regulation of CMV release remains underexplored, though mutations in *covS*, Δ*sigB*, and *Pst/SenX3-RegX3* genes have been implicated in BEV biogenesis. Furthermore, Rath et al. also demonstrated that inactivation of the virR gene leads to hypervesiculation in *M. tuberculosis* [85,86,87,88]. However, despite the knowledge already acquired, further research is needed to elucidate the precise mechanisms governing CMV release in Gram-positive bacteria [26].

**Table 2:** Characteristics of bacteria-derived extracellular vesicles (BEVs).

BEV Type	Producing Bacteria	Size	Origin/Composition	Biogenesis Mechanism	Functions	References
Outer membrane vesicles (OMVs)	Gram-negative	20–400 nm	Outer membrane: LPSs, proteins, and virulence factors.	Outer membrane budding, phospholipid accumulation, and peptidoglycan linkage loss	Virulence factor transport, HGT, biofilm formation, and host immune modulation	[70,71,78]
		10–300 nm	Outer membrane, periplasmic components	OM–PE bond loss; PE fragment accumulation; membrane curvature	Cellular communication, surface modification, waste removal	[13,28,77]
		Variable	Cytoplasmic materials (DNA, ATP)	Similar to OMVs	Genetic material transfer	[29,79]
Explosive outer membrane vesicles (E-OMVs)	Gram-negative	Variable	Outer membrane	Phage-mediated lysis	Genetic material transfer	[81]
Outer-inner membrane vesicles (O-IMVs)	Gram-negative bacteria	Variable; often larger than OMVs	Enclosed by both outer and inner membranes; contain cytoplasmic components	Bulging of both inner and outer membranes, through cell wall degradation or membrane fusion events	Transfer of intracellular components, including genetic material; potential role in stress response	[74]
Cytoplasmic membrane vesicles (CMVs)	Gram-positive	20–400 nm	Cytoplasmic membrane, cross peptidoglycan layer	Turgor pressure; local peptidoglycan degradation by EV-associated enzymes (PBPs, autolysins)	Communication, immune stimulation, genetic exchange	[29,73,75,82,83,84,85,87]
Tube-shaped membranous structures (TSMSs)	Gram-positive and Gram-negative	50–70 nm	Tubular, intercellular bridges	Poorly understood	Intercellular material exchange	[26,45,72,80,82]
Tuberculosis vesicles (TBVs)	Mycobacterium tuberculosis	Variable	Enriched with immunomodulatory molecules	Genetic regulation by Pst/SenX3-RegX3 and vesiculogenesis pathways	Modulate host immune responses, potential role in latency and persistence	[86,88]

## 5. Eukaryotic EVs

Eukaryotes share significant similarities with archaea in fundamental biological processes such as translation and transcription. These findings have sparked intense debates among researchers regarding the evolutionary relationship between archaea and eukaryotes. Some researchers argue that eukaryotes originated from archaea, while others propose that both groups share a common ancestor [89,90,91]. Regardless of these perspectives, all eukaryotes exhibit a bacterial legacy, representing a fusion of archaeal and bacterial characteristics while maintaining unique eukaryotic features [1,89].

Eukaryotic extracellular vesicles (EVs) are classified into exosomes, microvesicles (MVs), and apoptotic bodies based on their cellular origin, biogenesis pathways, size, and functions [4,92,93]. Each of these subtypes has a high therapeutic potential because they may function as messengers in both physiological and pathological conditions. These subtypes hold significant therapeutic potential as mediators in physiological and pathological processes.

### 5.1. Exosomes

Exosomes originate from the endosomal system, range from 30 to 150 nm in size, and are characterized by specific surface protein markers, including tetraspanins (CD9, CD63, and CD81), heat shock proteins (Hsp70 and Hsp90), MHC molecules, and proteins involved in multivesicular body (MVB) biogenesis (e.g., TSG101 and ALIX) [3]. Notably, they are specific to eukaryotic cells [94,95]. The word “exosome” was initially used for vesicles released from the plasma membrane but later became specific to intraluminal vesicles (ILVs) formed within MVBs and released upon MVB fusion with the plasma membrane [96] (Table 3).

Exosomes are secreted by all cell types and have been detected in various biological fluids, including plasma, urine, semen, saliva, bronchial fluid, cerebrospinal fluid, breast milk, serum, amniotic fluid, synovial fluid, tears, lymph, bile, and gastric acid [97].

Exosome formation occurs in three stages: (i) plasma membrane invagination leading to endosome formation, (ii) invagination of the endosomal membrane, forming MVBs containing ILVs (true exosomes) [98], and (iii) MVB fusion with the plasma membrane, followed by exosome release [94]. Exosome release follows two main pathways: one dependent on the ESCRT complex (endosomal sorting complex required for transport) [99,100], and one independent of ESCRT [101,102]. In this case, the latter pathway relies on sphingomyelinase and tetraspanins [100,102,103].

### 5.2. Microvesicles

Microvesicles (MVs) form through outward budding of the plasma membrane [104]. MVs are larger than exosomes (100–1000 nm) and are released via a calcium-dependent mechanism, exhibiting similarities to EVs produced by certain monoderm bacteria and archaea [1] (Table 4).

A hallmark of MVs is the presence of phosphatidylserine on their outer membrane. Additionally, MVs are characterized by proteins associated with lipid rafts, which are microdomains of the membrane rich in cholesterol, sphingolipids, and proteins responsible for signal transduction, membrane trafficking, or reorganization of the cytoskeleton. Surface glycan-binding proteins on MVs may be crucial for targeting and cellular interactions. Initially, as with exosomes, MVs were considered a cellular waste disposal mechanism. However, further studies revealed their role in cell–cell communication, facilitating interactions between neighboring and distant cells, similar to exosomes [93].

### 5.3. Apoptotic Bodies

Apoptotic bodies (ApoBs) share several structural and functional characteristics with exosomes, although they are significantly larger, with diameters ranging from 500 nm to 2 µm [105,106]. These vesicles originate from apoptotic cells through a tightly regulated process characterized by chromatin condensation, plasma membrane blebbing, and subsequent cellular fragmentation into membrane-bound structures [106]. This process is distinct from the biogenesis of exosomes, which are generated through the endosomal pathway and typically range in size from 30 to 100 nm [95] (Table 5).

Functionally, ApoBs serve as a critical mediator of intercellular communication. Like exosomes and microvesicles, they modulate the activity of professional phagocytes and non-phagocytic neighboring cells, thereby contributing to tissue homeostasis. Apoptotic bodies are not merely cellular debris but serve as active vectors for the transfer of biological molecules, including DNA, peptides, and oxidized phospholipids, influencing the differentiation and proliferation of endothelial progenitor cells (EPCs) [107]. Their intravesicular content depends on their cell of origin and can include proteins, sugars, lipids, adhesion integrins, growth factors, protease inhibitors, and different types of genetic material such as double-stranded DNA, mRNA, or microRNA [105]. This function highlights their pivotal role in vascular repair and regeneration, as they have been shown to enhance EPC recruitment and differentiation [107,108].

Despite these functional similarities with exosomes, ApoBs exhibit a more selective range of target cells. This selectivity suggests a distinct role in apoptotic cell clearance and in the regulation of signaling pathways involved in both physiological and pathological processes. The specific uptake of apoptotic bodies by certain cell types, such as endothelial progenitor cells, contributes to vascular homeostasis, potentially serving as a signaling mechanism in response to endothelial injury [107]. Moreover, their role in immune regulation has been increasingly recognized, with evidence suggesting that ApoBs may influence inflammatory responses and immune cell activation [95,105,108]. The presence of apoptotic bodies in various biological fluids, including blood, plasma, cerebrospinal fluid, and saliva, further supports their importance as extracellular messengers with diverse physiological roles [105]. This distinction underscores the need for further investigation into the molecular mechanisms governing ApoB function and their broader implications in intercellular signaling and immune modulation.

## 6. Interdomain Interactions

### 6.1. Role in Diseases

EVs from archaea appear to play a vital role in disease pathogenesis modulation and interactions with other domains. However, despite their promising importance, knowledge remains limited due to the challenges in studying archaea. Here are some studies that described the potential of their intervention in the progress of a disease and evolutionary dynamics (Table 6).

Acute myocardial infarction represents one of the major complications of atherosclerosis and often results from the rupture of atheromatous plaques. A variety of pathogens have been involved in human ruptured thrombosed atherosclerotic plaques; these include *Chlamydophila pneumoniae* and *Mycoplasma pneumoniae*. Where *M. pneumoniae* and archaea symbiosis occurs in susceptible plaques, it has been observed to promote infectious vesicle release, thereby weakening the protective function of the exosomes. These exosomes often engulf and degrade infectious antigens to protect against plaque rupture. The evidence is presented that EVs in vulnerable plaques are double membrane-bound structures with electron-lucent content containing archaeal DNA associated with myxomatous degeneration [109,110].

Archaea may facilitate the survival and proliferation of chlamydia and mycoplasma at the vulnerable atherosclerotic plaques. This facilitation occurs through mechanisms such as the production of Metalloproteinase and the release of EVs that may play a role in the lack of protection of exosomes [111,112].

Interactions between archaea and eukarya, in which EVs play a significant role in human disease, have been reported in Chagas disease-related heart failure. The presence of EVs containing archaeal DNA was found to be increased in *T. cruzi*-infected patients who developed heart failure compared to those with asymptomatic conditions. The differing outcomes appear to be driven not by *T. cruzi* itself, but rather by the symbiotic archaea and their vesicles [113].

### 6.2. Exploring the Potential Role of EV Cross-Talking

Until now, the exact role played by EVs in the cross-talk between archaea and other domains has not been well characterized. Various variables include a knowledge gap in this area. Though archaea have broad distributions in environments including ocean water, surface soil layers, marine subsurface sediments, and mesophilic niches such as the human oral cavity, gut, and vagina, most of the traditionally studied archaea live in extremely hot environments, such as hot springs, salt lakes, and submarine volcanic habitats, hostile to the other cell types. These environmental niches limit the potential interactions between archaea and other domains. Moreover, the culture of the great majority of archaea species is complicated under laboratory conditions and may lead to the inability to get complete information about this domain [114,115] (Table 7).

Examining organismal evolution and ecology reveals a dynamic landscape shaped by ancient genetic exchanges and ongoing interactions between archaea and other domains of life.

An interesting analysis of the reductive citric acid cycle (rTCA), crucial to the metabolism of the most ancient organisms, reveals fascinating insights into the adaptive strategies employed by microorganisms. It shows that actinobacteria and proteobacteria, despite being very distant from the root of the phylogenetic tree, acquired the genes associated with the rTCA cycle, which are found in several organisms of the archaeal kingdom. This suggested that horizontal gene transfer (HGT) may serve as a mechanism for acquiring genes in an adaptive strategy for survival in diverse environments by breaching host barriers [116].

The distribution of the M32 family of peptidases across bacteria, archaea, and trypanosomatids provides further evidence for the widespread occurrence of HGT. Sequence alignment studies suggest that these genes had been acquired through HGT between an ancestral proteobacterium and an ancestral trypanosomatid. This hypothesis would explain the presence of M32 genes in trypanosomatids and their absence in other eukaryotic organisms [117]. It is important to note that while it is not clear whether these HGT events occurred through extracellular vesicles or other mechanisms, these examples are nonetheless valuable in illustrating the potential impact of EV-mediated gene transfer. Such gene transfer events provide receiving organisms advantageous traits, driving the evolutionary process.

Due to their wide distribution, it is believed that most archaea and bacteria also interact at the metabolic level. This occurs everywhere in nature, including the human body, where this cooperation may facilitate disease, raising the question of whether some archaea can be considered potential human pathogens [118].

However, understanding this interaction is limited by the difficulties of recreating in experimental conditions environments such as the natural ecosystem in which bacteria and archaea cooperate in a mutualism and syntrophic process [119].

## 7. Conclusions

In summary, this review gives an overview of archaea’s interaction with other life domains, pointing out the possible role of EVs in facilitating the communication and exchange of biomolecules between archaeal cells and other cellular entities. This is an insight into horizontal gene transfer mechanisms, particularly the acquisition of metabolic pathways and genetic elements that have lit up the dynamic nature of microbial evolution. This review’s objective ranges from researching EVs that are produced by bacteria, eukaryotes, and archaea, focused on archaeal EVs and their possible involvement in cross-domain communication, to a comprehensive discussion on the structure and biogenesis of bacterial extracellular vesicles—be it the complexities of BEV formation across different organisms or the distinctions between eukaryotic EVs, such as exosomes, microvesicles, and apoptotic bodies, and BEVs. However, there are still profound knowledge gaps in specific mechanisms by which EVs from archaea mediate communication between other domains and might, to a certain extent, participate in the development of human diseases. Although several studies have suggested that EV-mediated HGT, also known as “vesiduction,” is a driver of microbial evolution, much about the biogenesis and function of EVs in archaea, including their interactions with other domains of life and involvement in pathogenesis, remains uncharacterized. These are, therefore, some of the gaps that need to be investigated in the future, as understanding the role of archaeal EVs might provide new insights into microbial ecology and the evolution of interdomain communications, thus ultimately providing new therapeutic approaches toward the pathologies in which they are involved. Further investigation is also invited in the comparative biogenesis of EVs among the different domains, which information is available, and their potential applications in biotechnology and medicine.

## Figures and Tables

**Table 1:** Functional roles and mechanisms of extracellular vesicles (EVs) in archaea.

Domain/Order	Role of Extracellular Vesicles (EVs)	Molecular Mechanisms	References
*Euryarchaeota: Thermococcales*	Stabilization of extracellular DNA under hyperthermic conditions; protection from nucleolytic degradation (e.g., micrococcal nuclease)	-Encapsulation of DNA within EVs for protection against degradation -Fusion of EVs with recipient cells for genetic transfer	[48,54,55,56]
*Euryarchaeota:* *Halobacteriales*	Facilitation of genetic recombination between viral, plasmid, and host chromosomal DNA elements	-Transport and delivery of recombinant DNA via EVs -Mediation of horizontal gene transfer (HGT) between cells	[48,54,55,56]
*Crenarchaeota: Sulfolobales*	Mediation of horizontal gene transfer (HGT) via EVs, a process termed ”vesiduction”	-Release of EVs containing nucleic acids -EV adhesion to recipient cells, followed by DNA internalization into the cytoplasm	[56,57,58,59]
*Haloarchaea* (e.g., *Haloarchaea volcanii*)	Extracellular RNA packaging for regulation of gene expression at the population level	-Active packaging of RNA into EVs for population-wide gene regulation -EV-mediated intercellular communication	[56,57,58,59]
*Thermococcus* spp. *(T. prieurii* and *T. kodakaraensis)*	Detoxification by sequestration and extracellular expulsion of excess sulfur	-Formation of sulfur-enriched EVs -Excretion of sulfur metabolites to alleviate cellular toxicity	[60]
*Crenarchaeota: Sulfolobus islandicus*	Transfer of carbon and nitrogen for supporting heterotrophic growth under nutrient-limited conditions	-Release of EVs containing lipids, proteins, and nucleic acids -Metabolic support to heterotrophic microorganisms through EVs	[20,61]
DPANN: *Nanoarchaeum equitans* and *Ignicoccus hospitalis*	Metabolite exchange in symbiotic relationships; involvement of EVs in host cell division	-Periplasmic transport of metabolites via EVs -Contribution of EVs to the formation of the outer membrane during cell division	[62,63,64,65,66,67,68,69]

**Table 3:** Exosomes’ key features.

Features	Description	References
General characteristics	Origin: endosomal system in eukaryotic cells.	[3,94,95]
	Size: 30–150 nm in diameter.	
	Surface markers: CD9, CD63, CD81, Hsp70, Hsp90, MHC molecules, TSG101, and ALIX.	
Presence in fluids	Detected in plasma, urine, saliva, cerebrospinal fluid, and breast milk	[97]
Formation process	(1) Endosome formation via membrane invagination.	[94,96,98]
	(2) ILVs develop within MVBs.	
	(3) MVBs fuse with the plasma membrane, releasing exosomes.	
Biogenesis and release	Pathways: ESCRT-dependent and ESCRT-independent (lipid rafts, ceramides, and tetraspanins).	[99,100,101,102,103]
	Release mechanism: controlled by sphingomyelinase and tetraspanins.	

**Table 4:** Microvesicles’ key features.

Characteristics	Description	References
Biogenesis mechanism	Formed via outward budding of the plasma membrane, regulated by a calcium-dependent process	[104]
Size range	100–1000 nm	
Membrane composition	Enriched with phosphatidylserine on the external leaflet, a defining feature of MVs	
Surface glycan-binding proteins	Glycan-binding proteins on the MV surface mediate cell targeting and intercellular interactions	
Biological functions	Intercellular communication	[93]
Evolutionary analogs	Share structural and functional characteristics with extracellular vesicles from monoderm bacteria and archaea	[1]

**Table 5:** Apoptotic bodies’ key features.

Characteristics	Description	References
Function in cell communication	Apoptotic bodies transfer genetic material and proteins, playing a crucial role in homeostasis.	[105]
Discovery and terminology	First described as a key process in cell turnover, development, and tumor regression.	[106]
Comparison with exosomes	Larger than exosomes, apoptotic bodies result from cell disassembly and aid immune signaling.	[95]
Role in endothelial repair	Apoptotic bodies enhance endothelial progenitor cell differentiation, aiding vascular repair.	[107]
Study in emerging models	Research on nontraditional models expands understanding of apoptosis beyond classic systems.	[108]

**Table 6:** Archaeal EVs in disease.

Pathogen/Disease	Location of Archaeal EVs	Outcomes	Reference
*Chlamydophila pneumoniae & Mycoplasma pneumoniae*	Atherosclerotic plaques	Associated with inflammation and acute myocardial infarction.	[109]
	Serum (measured via serological study)	High serum antibody titers correlated with acute coronary syndromes.	[110]
	Vulnerable plaques (myxoid matrix, foam cells)	Archaea contribute to plaque instability and co-infections.	[111]
*Mycoplasma pneumoniae*	Serum (extracellular vesicles from severe AMI patients)	Elevated archaeal EVs linked to worse prognosis in severe AMI.	[112]
Chagas disease	Serum (microvesicles in Chagas disease patients)	Archaea in microvesicles may contribute to heart failure in Chagas disease.	[113]

**Table 7:** EV interactions between archaea and other domains.

Topic	Key Points	References
Role of EVs in interdomain interactions	-EVs may facilitate horizontal gene transfer (HGT).	[114,115,116,117]
	-Acquisition of adaptive traits and exchange of genetic material.	
	-Overcoming domain-specific barriers for gene transfer.	
EVs in metabolism and evolution	-The rTCA cycle may be influenced by gene transfer via EVs.	[116]
	-Facilitation of genetic exchange between archaea and bacteria for environmental adaptation.	
Horizontal gene transfer (HGT)	-Mediation of gene transfer (e.g., M32 peptidase) between archaea, bacteria, and trypanosomatids.	[117]
	-Contribution to evolutionary diversification through the acquisition of new genes.	
Metabolic interactions between archaea and bacteria	-Facilitation of metabolic exchanges to improve ecosystem stability and cooperation.	[118]
	-Transfer of genes related to metabolism and pathogenicity, with potential links to human diseases.	
Challenges in studying EV-mediated interactions	-Difficulty in replicating natural conditions in the laboratory.	[119]
	-Complexity of natural mutualistic processes hindering experimental studies.	

## Data Availability

Data sharing is not applicable.

## Abbreviations

The following abbreviations are used in this manuscript:

EVs	Extracellular vesicles
BEVs	Bacteria-derived extracellular vesicles
AEVs	Archaea-derived extracellular vesicles
